# Research trends and scientific analysis of publications on burnout and compassion fatigue among healthcare providers

**DOI:** 10.1186/s12995-020-00274-z

**Published:** 2020-07-13

**Authors:** Waleed M. Sweileh

**Affiliations:** Department of Physiology, Pharmacology/Toxicology, College of Medicine and Health Sciences, An-Najah National University, Nablus, Palestine

**Keywords:** Burnout, Compassion fatigue, Research trends, Citation analysis, Research themes

## Abstract

**Background:**

Burnout and compassion fatigue are closely related concepts. Burnout is thought to develop from occupational stress while compassion fatigue results from being in a caregiver role leading to inability to get engaged in a caring relation. The objective of the current study was to investigate research trends, themes, citations, and key players of publications on burnout and compassion fatigue among healthcare providers.

**Methods:**

A validated search query was developed and implemented in the Scopus database. The study period was all times up to 2019.

**Results:**

Research query found 4416 publications. Publications started in 1978. Steep growth in the number of publications was observed in the last decade. There were seven research themes in the retrieved publications; six for burnout and one for compassion fatigue. Approximately 36% of the retrieved publications were about nurses; 36% were about physicians, 10% were about medical residents and the remaining did not include a specific profession. The retrieved publications received an average of 22.2 citations per article. Four of the top 10 active journals were in the field of nursing and two in the field of general medicine, one in public health, one in neurology, one in psychology, and one was multidisciplinary. The USA ranked first with 1292 (29.3%) articles followed by Spain (*n* = 248; 5.6%) and the UK (*n* = 247; 5.6%). Mayo Clinic was the most active institution (*n* = 93; 2.1%) followed by Harvard University (*n* = 46; 1.0%) and University of Washington, Seattle (*n* = 45; 1.0%). A total of 16,108 authors participated in publishing the retrieved documents, an average of 3.6 authors per article. *Shanafelt, T.D.* was the most active author (*n* = 78; 1.8%) followed by *Dyrbye, L.N.* (*n* = 43; 1.0%), and *West, C.P.* (*n* = 37; 0.8%). A total of 472 (10.7%) articles declared funding.

**Conclusion:**

The current study was carried out to draw attention to the wellbeing of healthcare providers. Retrieved literature was dominated by high-income countries. Lack of information from low- and middle-income countries will hinder planning for interventional strategies and will negatively affect the health system and the patients. Health researchers in low- and middle-income countries need to focus on burn out and compassion fatigue.

## Background

Burnout (BO), the opposite of wellbeing, is defined as an emotional and behavioral impairment in response to the prolonged and high level of exposure to occupational stress [1, 2]. Burnout encompasses feelings of emotional exhaustion, depersonalization, and reduced professional efficacy [1, 3]. Several national studies indicated that BO has reached high levels among healthcare providers and resulted in documented negative effects on patients’ health [4–9]. Nurses are known to be at higher risk of developing BO than other professions due to high responsibilities and work demand [10, 11]. Compassion fatigue (CF) has been defined as a state of physical or psychological distress in caregivers or rescuers, which occurs as a consequence of an ongoing and snowballing process in a demanding relationship with needy individuals [12]. Burnout and CF are closely related and overlapping. A systematic review of the prevalence of BO and CF among professionals in intensive care units found a wide range of prevalence data among different studies due to lack of unity in measurement and “lack of common understanding of the theoretical constructs, which is reflected by the variously defined (and interpreted) negative outcomes of providing care in the ICU setting among the included studies” [13]. The findings of the systematic review made the door open for the true prevalence of BO and CF in published studies due to the complexities in measuring and defining the outcome of emotional distress. However, it is believed that BO and CF have different underlying mechanisms. Burnout is thought to develop from occupational factors that could lead to lack of enthusiasm and productivity, whereas CF results unknowingly and from being in a caregiver role leading to inability to get engaged in a caring relation [14, 15].

Physicians, nurses, and other healthcare providers are subject to high levels of occupational and psychological stress. For example, healthcare providers have to deal with death issues, to observe patients’ sufferings, to cope up with the demands of patients’ families, to make ethical decisions, to make critical and quick life-saving decisions, to adjust and endure the long working hours and night shifts, and to adapt to under-staffing or conflict issues in a certain country [9, 16–20]. The US Critical Care Societies Collaborative (CCSC) acknowledged the importance of BO and other psychological disorders in critical care health-care professionals and published a statement calling for publications that would focus attention on this issue to (1) raise awareness of BO within the critical care community and (2) inform multiple stakeholders of their potential roles in reducing BO and its negative consequences in health-care professionals and their critically ill patients [21]. Investing in the wellbeing of healthcare providers is important because BO and CF negatively affect all components of the health system including patient satisfaction and health cost [4, 5, 7, 22–25].

A substantial volume of literature on BO and CF has been published in a wide range of jobs and professions including nursing, physicians, residents, neurologists, psychotherapists, surgeons, orthopedics, paramedics, and others. According to the Global Citizen website, investing in healthcare workers is one of 13 most pressing global health issues for the coming decade [26]. The World Health Organization (WHO) is stimulating countries to invest in healthcare workers and improve their life. Therefore, it is very important to carry out a scientific analysis of BO and CF-related literature among healthcare providers since they are at the frontline in the health system especially at times of emergencies or outbreaks. The scientific analysis of a certain topic focuses on the growth of publications, research themes, important debatable issues, and key players in that topic. This analysis is important for health policymakers, academics, researchers, and international health organizations to implement preventive strategies. The objective of the current study was to analyze literature on BO and CF among healthcare providers using a large and powerful database. In specific, research trends, research themes, active key players, and citations were analyzed and presented.

## Method

### Database

SciVerse Scopus, owned by Elsevier, was used for the merits it has over other databases. Scopus is the largest scientific database available. It has more than 23,000 indexed journals in all disciplines. It is 100% inclusive of Pubmed and it has almost double the number of journals indexed in Web of Science [27]. Scopus allows the export of data to Microsoft Excel and other programs such as VOSviewer program [28] which is a mapping program available as a free on-line program. Scopus has many functions that allow for information management. It can do a citation analysis of the retrieved literature. Scopus has been used in many previously published studies to assess research trends and growth pattern of many medical topics [29–31].

### Search query

The search query was developed based on keywords related to BO and CF with keywords related to healthcare workers/professionals. The search was carried out using the title search to avoid irrelevant publications. The keywords used were obtained from the basic definitions of BO and CF. Examples of keywords related to BO and CF included the followings: “burnout”, “burn - out”, “compassion fatigue”, “burning out”, “burn out”, “occupational stress”, “professional *stress”, “emotional *stress”, “emotional exhaustion”, “secondary trauma*”, “vicarious trauma*”, “psychological *stress”, “empathy”, “physical exhaust*”, “depersonalization”, “feeling* of cynicism”, detachment, “depletion of energy”, “wellbeing”. The quotation marks were used to indicate the specific keyword or phrase while asterisk was used as a wild card to retrieve similar terms. Examples of keywords or phrases related to healthcare providers included: health*, nurs*, physician, doctor, pharmac*, “healthcare”, “medical”, resident, clinician*, paramedic, hospital, “ICU”, emergency, surgeon, orthopedic, residency, surgical, oncology, neurology, neurologist, “medicine”, oncologist, psychiatrist, midwife, clinic. These keywords were followed by an exclusion step. In the current study, all documents on medical or nursing students were excluded since the focus was on healthcare providers and medical or nursing students are not yet an official healthcare provider. The current study was limited to journal publications. Therefore, books, book chapters, and conference abstracts were all excluded. The study period was defined as all times until 2020. Supplementary material 1 is a flow chart of the search query while Supplementary material 2 is the exact keywords used in each step in the flow chart.

### Validation

The search query was finalized based on certain validation criteria. Therefore, the search query was fine-tuned until the validation criteria were met. The criteria included the absence of any false-positive (irrelevant) publication in the top 200 cited publications and the absence of false-negative (missing publications) publications. The search query utilized title search and therefore the presence of false-positive results was minimum. The use of certain phrases such as “depletion of energy” was associated with certain false-positive results. To avoid this, a certain constraint was added with the phrase “depletion of energy”. The constraint was the presence of the keyword “burnout” or “compassion fatigue” in the abstract of the publications having the phrase “depletion of energy” in the title. Validation for the absence of false-negative results (missing data) was carried out using the profile of most active authors in this field. In the current study, *Shanafelt, T.D*.; *Dyrbye, L.N*.; and *West, C.P*. were among top 10 active authors and were used in the validation step by comparing the number of publications retrieved for each one of them (78; 43; and 37 respectively) with the actual number of relevant publications present in their personal Scopus profile. This process was carried for the top 10 active authors and yielded a significant and strong correlation (*p* = 0.003; *r* = 0.97) between the number of publications retrieved and the actual numbers present in active authors’ profiles. This validation step was adopted from previously published articles on research analysis [32].

### Export

The validated search query was applied in the advanced search function using appropriate Boolean operators and the retrieved publications were exported to programs used for data analysis and presentation. The exported data to Microsoft Excel included annual growth of publications, subject areas of the retrieved publications, types of publications, languages, countries involved in publishing the retrieved publications, journals, authors, institutions, funding, and citations. A “CSV” file of all publications was exported from Scopus to VOSviewer for mapping purposes.

### Scientific indicators

The following scientific indicators were presented: the annual growth of publications, the top 10 active countries, the top 10 active institutions, the top 10 active authors, the top 10 cited publications, map of most frequent terms and author keywords, and top 10 active journals.

## Results

### Volume and types of publications

The search query found 4416 publications. There were eight different types of documents in the retrieved publications. Research articles (*n* = 3564; 80.7%) was the most common type followed by review articles (*n* = 329; 7.5%), letters (*n* = 195, 4.4%), notes (*n* = 169; 3.8%), editorials (*n* = 109; 2.5%), and conference papers (*n* = 50; 1.1%). Of the 33 different languages encountered in the retrieved publications, English was the most common (*n* = 3710; 84.0%) followed by Spanish (*n* = 226; 5.1%), French (*n* = 107; 2.4%), German (*n* = 103; 2.3%), Portuguese (*n* = 79; 1.8%), and Italian (*n* = 61; 1.4%).

### Subject areas of the retrieved publications

Most of the retrieved documents were published within the subject area of medicine (*n* = 3042; 68.9%) followed by nursing subject area (*n* = 1070; 24.2%), psychology subject area (*n* = 510; 11.5%), social sciences (*n* = 374; 8.5%), biochemistry/genetics/molecular biology (*n* = 139; 3.1%), and business, management and accounting (*n* = 103; 2.3%).

### Evolution and growth of publications

**Fig. 1:**
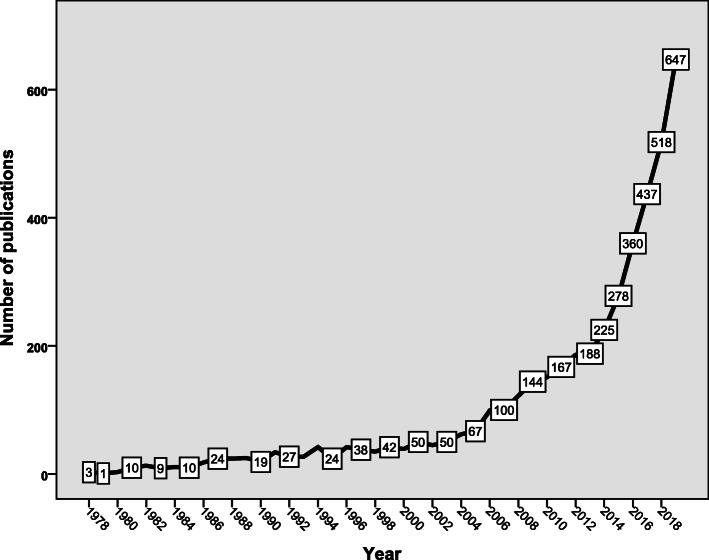
Annual growth of publications on burnout and compassion fatigue among healthcare providers

The retrieved publications started in the late 1970s. The oldest retrieved articles were published in 1978 [33, 34]. The annual number of publications remained below 100 publications per year until 2008 then showed a steep rise in the number of publications (Fig. 1).

### Most frequent author keywords (research themes)

**Fig. 2:**
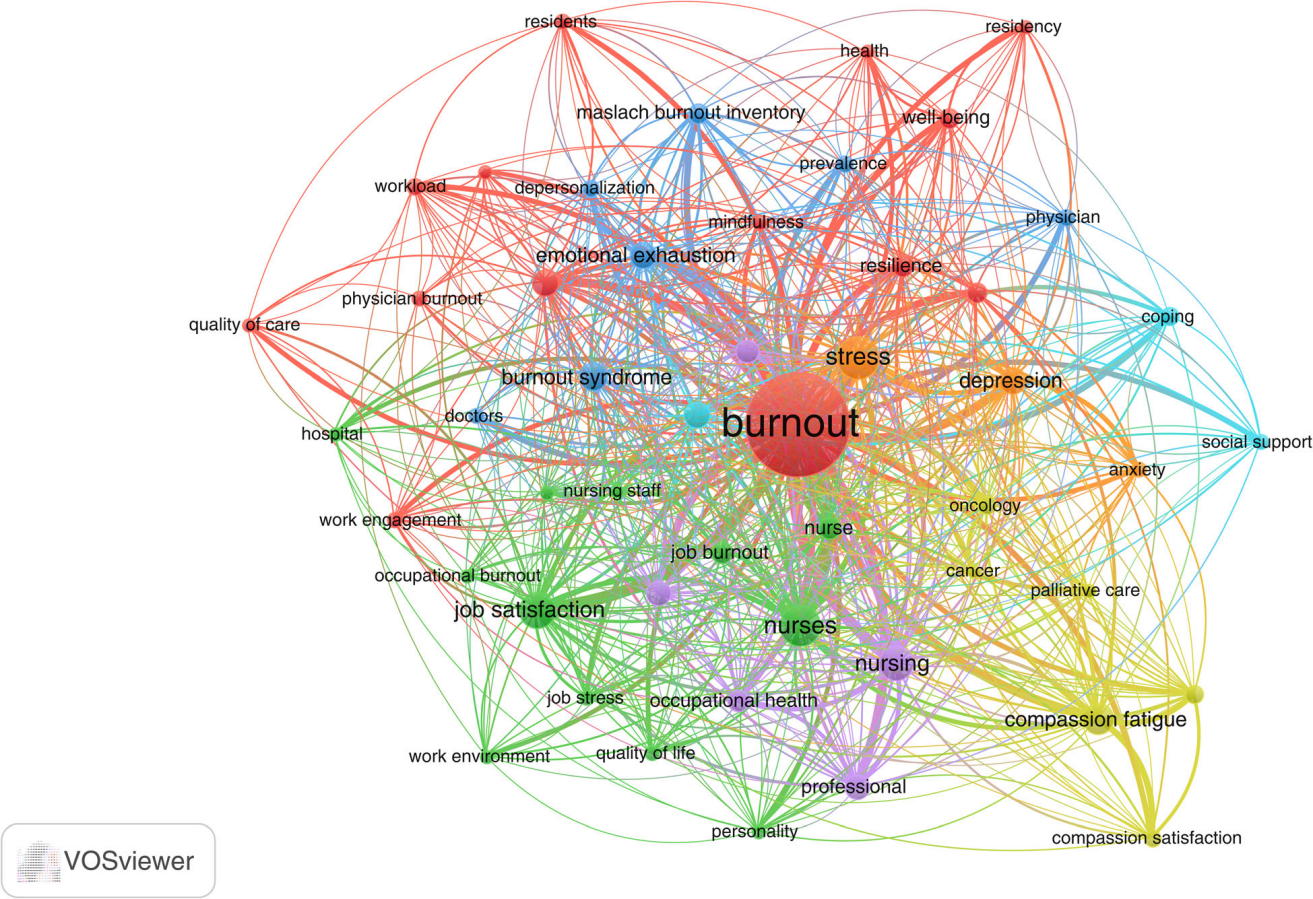
Network visualization map of author keywords. Each color represents a cluster of related author keywords (research theme/topic)

Author keywords with a minimum occurrence of 50 times were mapped (Fig. 2). The keyword “burnout” was in the center of the map and had the highest occurrence (*n* = 2133). The map showed seven overlapping clusters representing specific research themes in the retrieved publications. The clusters included a research theme (yellowish-green cluster) about compassion fatigue, palliative care, cancer, and oncology. A second research theme (light blue) was about occupational stress, coping, and social support. The third research theme (orange) was about stress, anxiety and depression. The fourth research themes (red) was the largest and focused on burnout, resilience, mindfulness, wellbeing, empathy, residents, and quality of care. The fifth theme (blue) was about the physician’s burnout and Maslach Burnout Inventory (MBI). The sixth theme (light purple) focused on professional burnout, nursing, and mental health. The seventh theme (green) focused on burnout, nursing, personality, job satisfaction, and quality of life.

### Types of healthcare professions encountered

Based on title analysis, the retrieved publications included 1594 (36.0%) documents about nurses/nursing, 1634 (37.0%) about physicians/doctors/clinicians, 418 (9.5%) about medical residents, 42 (1.0%) about pharmacists/paramedics, and 768 (17.4%) about healthcare workers/providers/staff/employees in general and without reference to a specific profession. There was an overlap in certain documents because they discussed BO and CF among more than one profession. The total number of documents based on the title search for professions did not add up to 4416. There were at least 106 (2.4%) documents in which the title did not have any of the professions or terms listed above.

### Top ten cited documents

The retrieved publications received 98,016 citations, an average of 22.2 citations per article. The top-cited article was published in the *Journal of the American Medical Association* (JAMA) in 2002 [22]. The article concluded that “ In hospitals with high patient-to-nurse ratios, surgical patients experience higher risk-adjusted 30-day mortality and failure-to-rescue rates, and nurses are more likely to experience burnout and job dissatisfaction”. The second top-cited article was published in 2002 in *Annals of Internal Medicine* and concluded that “Burnout was common among resident physicians and was associated with self-reported suboptimal patient care practices” [6]. The top 10 cited articles included two articles about BO among nurses, three articles about medical residents, two about surgeons, and three about physicians (Table 1). Four of the top 10 cited articles were published in *JAMA* and two were published in *Annals of Surgery*.

**Table 1:** Top ten cited documents on burnout and compassion fatigue among healthcare providers [4–6, 8, 22, 25, 35–38]

Rank	Title	Year	Source title	Cited by
1	Hospital nurse staffing and patient mortality, nurse burnout, and job dissatisfaction	2002	Journal of the American Medical Association	2871
2	Burnout and self-reported patient care in an internal medicine residency program	2002	Annals of Internal Medicine	1118
3	Burnout and satisfaction with work-life balance among US physicians relative to the general US population	2012	Archives of Internal Medicine	1107
4	Association of an educational program in mindful communication with burnout, empathy, and attitudes among primary care physicians	2009	JAMA - Journal of the American Medical Association	782
5	Changes in Burnout and Satisfaction with Work-Life Balance in Physicians and the General US Working Population between 2011 and 2014	2015	Mayo Clinic Proceedings	754
6	Association of perceived medical errors with resident distress and empathy: A prospective longitudinal study	2006	Journal of the American Medical Association	695
7	Burnout and medical errors among American surgeons	2010	Annals of Surgery	628
8	Nurse burnout and patient satisfaction.	2004	Medical care	522
9	Resident burnout	2004	Journal of the American Medical Association	481
10	Burnout and career satisfaction among American surgeons	2009	Annals of Surgery	454

### Top ten active journals

**Fig. 3:**
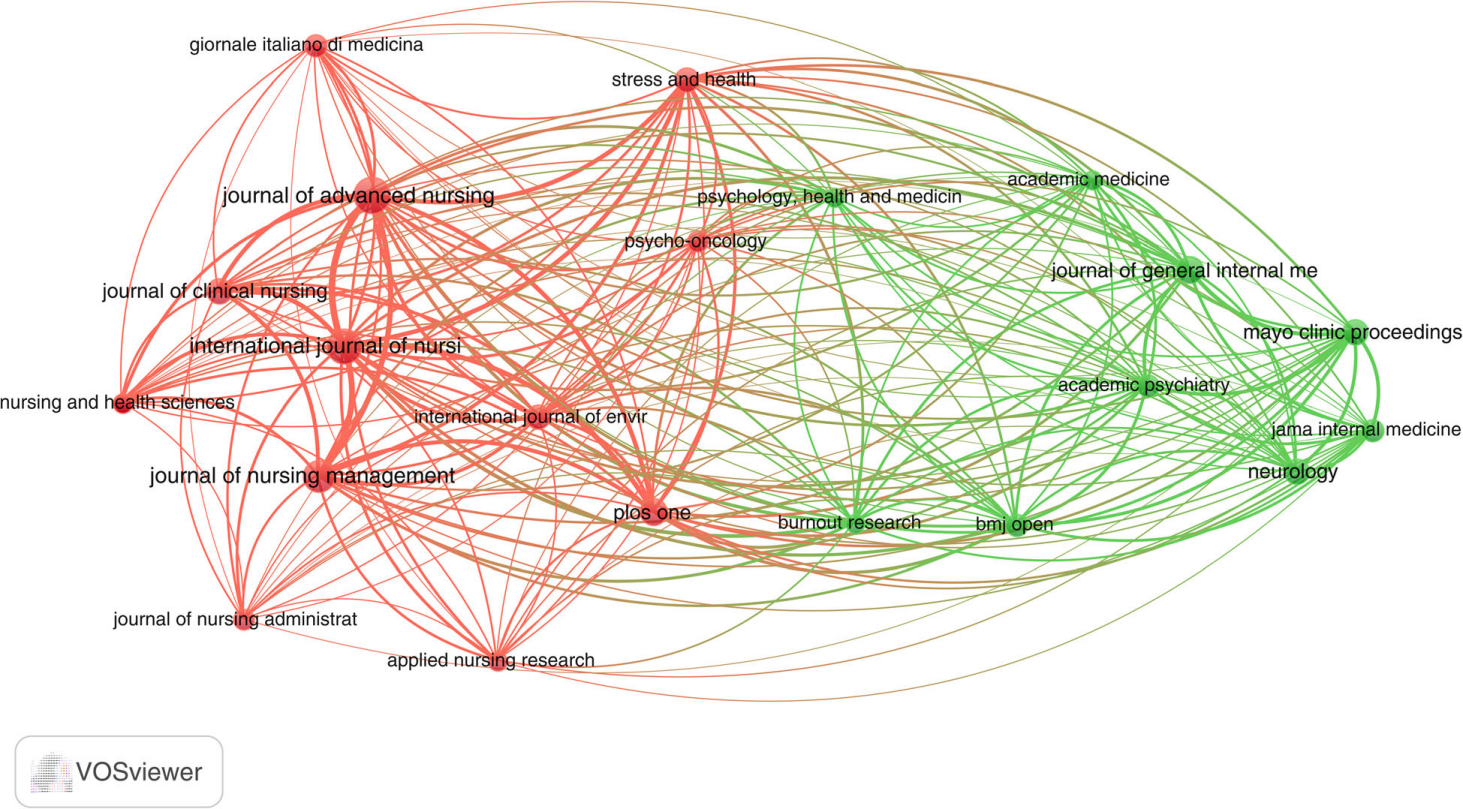
Network visualization of bibliographic coupling of documents published in journals with minimum research output of 20 articles. Journal name was limited to 30 characters. Therefore, few journal names were truncated

The retrieved publications were published in 1632 different journals. The top 10 active journals were listed in Table 2. The top active journal was *Journal of Advanced Nursing* (*n* = 65; 1.5%). Four of the top 10 active journals were in the field of nursing, two in the field of general medicine, one in public health, one in neurology, one in psychology, and one was multidisciplinary. Mapping active journals for bibliographic coupling showed that articles published in journals related to neurology, psychiatry, psychology, and medicine had similar citation pattern while articles published in journals related to nursing, public health, and psycho-oncology had similar citation pattern (Fig. 3).

**Table 2:** Top ten active journal in publishing documents on burnout and compassion fatigue among healthcare providers

Rank^**a**^	Journal Name	Frequency	% ***N*** = 4416
1	*Journal of Advanced Nursing*	65	1.5
2	*International Journal of Nursing Studies*	56	1.3
3	*Journal of Nursing Management*	48	1.1
4	*Journal of General Internal Medicine*	37	0.8
5	*Plos One*	34	0.8
6	*Journal of Clinical Nursing*	33	0.7
6	*Neurology*	33	0.7
8	*Mayo Clinic Proceedings*	32	0.7
9	*International Journal of Environmental Research and Public Health*	27	0.6
9	*Stress and Health*	27	0.6

^a^In ranking system equal values were given the same rank and one rank is skipped

### Top ten active countries

Authors in the top 10 active countries contributed to publishing 2868 (64.9%) articles. The USA ranked first with 1292 (29.3%) articles followed by Spain (*n* = 248; 5.6%) and the UK (*n* = 247; 5.6%). The top 10 active countries were distributed geographically in Northern America, Latin America, Europe, and the Western Pacific region (Table 3). The research activity was normalized by income and populations size, the USA ranked first followed by Brazil, China, and Turkey.

**Table 3:** Top ten active countries in publishing documents on burnout and compassion fatigue among healthcare providers

Rank	Institution	Frequency	% ***N*** = 4416	Number of publications per GDP per capita
1	United States	1292	29.3	20.5
2	Spain	248	5.6	8.1
3	United Kingdom	247	5.6	5.8
4	Canada	211	4.8	4.6
5	Australia	186	4.2	3.3
6	Italy	154	3.5	4.5
7	China	148	3.4	15.4
8	Brazil	144	3.3	16.0
9	Turkey	120	2.7	12.8
10	Germany	118	2.7	2.5

*GDP per capita* Gross Domestic Product (nominal) per capita obtained from World Bank data 2019

### Top ten active institutions

The top 10 active institutions were listed in Table 4. Mayo Clinic was the most active institution (*n* = 93; 2.1%) followed by Harvard University (*n* = 46; 1.0%) and University of Washington, Seattle (*n* = 45; 1.0%). The top active list of institutions included seven academic institutions and three clinical institutions (Mayo Clinic; Massachusetts General Hospital; Brigham and Women’s Hospital. Six of the top 10 active institutions were in the USA, two in Canada, one in Brazil, and one in Spain.

**Table 4:** Top ten active institutions in publishing documents on burnout and compassion fatigue among healthcare providers

Rank^**a**^	Institution	Frequency	% ***N*** = 4416	Country
1	*Mayo Clinic*	93	2.1	USA
2	*Harvard Medical School*	46	1.0	USA
3	*University of Washington, Seattle*	45	1.0	USA
4	*University of Toronto*	39	0.9	Canada
5	*Universidade de Sao Paulo - USP*	38	0.9	Brazil
6	*Universidad de Granada*	33	0.7	Spain
7	*Massachusetts General Hospital*	31	0.7	USA
8	*Western University*	30	0.7	Canada
9	*Brigham and Women’s Hospital*	29	0.7	USA
9	*University of California, San Francisco*	29	0.7	USA

^a^In ranking system equal values were given the same rank and one rank is skipped

### Authorship analysis

Sixteen thousand one hundred eight authors participated in publishing the retrieved documents, an average of 3.6 authors per publication. A total of 820 (18.6%) documents were single-authored. The top 10 active authors were listed in Table 5. *Shanafelt, T.D.* was the most active author (*n* = 78; 1.8%) followed by *Dyrbye, L.N.* (*n* = 43; 1.0%), and *West, C.P.* (*n* = 37; 0.8%). Six of the top 10 active authors were from the USA, two from Spain, one from Australia and one from the Netherlands.

**Table 5:** Top ten active institutions in publishing documents on burnout and compassion fatigue among healthcare providers

Rank^**a**^	Author	Frequency	% ***N*** = 4416	Country
1	*Shanafelt, T.D.*	78	1.8	USA
2	*Dyrbye, L.N.*	43	1.0	USA
3	*West, C.P.*	37	0.8	USA
4	*Cañadas-De la Fuente, G.A.*	19	0.4	USA
5	*Aiken, L.H.*	15	0.3	Spain
6	*Gómez-Urquiza, J.L.*	14	0.3	Australia
6	*Leiter, M.P.*	14	0.3	USA
6	*Linzer, M.*	14	0.3	USA
6	*Sloan, J.A.*	14	0.3	Netherlands
6	*Schaufeli, W.B.*	14	0.3	Spain

^a^In ranking system equal values were given the same rank and one rank is skipped

### Funding sponsors

A total of 472 (10.7%) published articles declared funding. The National Institutes of Health in the USA (*n* = 52; 1.2%) was the most active funding sponsor followed by Agency for Healthcare Research and Quality in the USA (*n* = 17; 0.4%), and Mayo Clinic in the USA (*n* = 13; 0.3%).

## Discussion

The current study aimed to analyze and assess scientific literature on BO and CF among healthcare providers. The study showed very steep growth in the last decade. More than one-third of the retrieved publications focused on nurses and nursing. Journals in the field of nursing also dominated the list of top 10 active journals. Authors and institutions in the USA had a leading role in this field followed by authors and institutions in Europe. The retrieved publications included several overlapping themes with CF in oncology being one distinct theme. The retrieved publications received a relatively high number of citations indicative of high visibility and importance.

The current study showed that publications started in 1978. The concept of BO emerged in 1974 by Herbert Freudenberger, an American psychologist [39]. The concept of BO in the retrieved literature appeared much earlier than the concept of CF which was introduced in the mid-1990s [12]. The current study showed a steep increase in the number of publications after 2005. The steep growth in the number of publications is, in part, due to the natural growth of science, increased number of researchers and the genuine demand to increase the efficiency of health services and patient satisfaction. Healthcare providers are required to do more than just patient care. A national survey on physician satisfaction and burnout revealed that 87% of physicians identified paperwork and other administrative activities as the leading cause of BO and stress [40]. The increased global health challenges such as antimicrobial resistance, infectious diseases outbreaks, hundreds of millions of refugees, and conflicts and wars in several parts of the world increased the pressure on healthcare workers which could be listed as one potential reason for increased publications in this field.

The current study showed the presence of several themes in the retrieved publications. One of the themes is the *Maslach Burnout Inventory* (MBI) used in assessing the prevalence of burnout. The MBI has been extensively used since its introduction in 1981 [2]. The MBI has shown excellent psychometric properties [41] and is now considered the standard measurement of BO. Compassion fatigue and satisfaction were a separate theme. Compassion is considered fundamental to nursing practice and failure to have compassion can lead to distress and decreased job satisfaction [42]. In the current study, compassion fatigue was represented by one theme while remaining six themes were about BO suggesting that literature on compassion fatigue was less than 15% (~ 660) of the total number of the retrieved publications. A recent study found 652 publications on CF using the Web of Science database and title/abstract search strategy [43]. Several studies have pointed out the limited number of publications on CF [15, 44]. A third important theme was coping strategies. Several approaches have been suggested to manage burnout among healthcare providers. The strategies include interventions at the individual and/or organizational levels [45, 46]. Intervention programs for reducing burnout at the individual level include a cognitive behavioral approach, coping skills and social support. The organization-directed programs focus on enhancing the work environment and decreasing job demands [39, 47, 48].

The current study indicated that approximately 36 and 46% of the retrieved publications were about nurses and physicians respectively. If we exclude publications on medical residents (~ 10.0%), the percentage of publications on burnout or CF among nurses and physicians would be equal (~ 36%). Studies on BO among nurses were mainly published in few specialized nursing journals. In contrast, documents on physicians were published in a wide range of journals including general medicine, health services, surgery, public health, psychiatry, and health management. This is one possible reason for the presence of four specialized nursing journals in the top 10 active journals.

The current study indicated that high-income countries in North America and Europe dominated the list of active countries, institutions, and authors. This was expected given the gap in research capabilities and funding between these countries and other ones in low- and middle-income category. The high number of publications in these countries does not mean that the prevalence of burnout or compassion fatigue among healthcare providers is higher than that in other countries. It might be the opposite. The work load of healthcare providers in low- and middle-income countries is high given the limited number of physicians and nurses in these countries. Furthermore, the lack or inadequate technology in some low- and middle-income countries adds the burden of documentation and follow up for healthcare providers in these countries. The limited resources and the volatile political and economic situations in many of these countries add up to the stress among healthcare workers in these countries. The limited number of publications from low- and middle-income countries could be attributed to lack of research experts, funding, or absence of an observatory health body concerned with the efficacy of the health system and satisfaction of the patient with reasonable cost [49–52].

In the current study, the average number of citations per document was higher than that reported for other medical topics such as epidermal parasitic skin disease [53], AIDS-related stigma [54], transgender health [55], and others. High number of citations is indicative of the importance and scientific impact of the topic. In fact. The content of the top-cited articles emphasized the importance and risk of BO and CF on patients and the health system due to increased medical errors [4, 5].

The current study gave an overview of BO and CF documents published in peer-reviewed journals. There are a few similar previously published documents. For example, a study on CF was carried out using Web of Science database [43] while another study focused on BO among ICU healthcare providers in Brazil [17]. The current study is unique in three aspects: (1) the use of the largest database available, Scopus; (2) the inclusion of both BO and CF in the analysis; and (3) the global nature of the analysis.

The current study has a few limitations. Despite the tremendous effort to include all relevant terms and the validation process, the presence of missing data or false-positive results remains a possibility. Efforts have been done to minimize errors through different validation approaches. There many health-related journals in low- and middle-income countries that are not indexed in Scopus. Therefore, the research activity from certain regions in the world was underestimated due to the limited number of national journals from those regions indexed in Scopus.

## Conclusion

The current study was carried out to analyze research trends, research themes, active key players, and citations of publications on BO and CF. The recent rapid growth in the number of publications and the high citation pattern are important signals for the necessity of intervention policies and programs at all levels to manage this issue. The idea that little information is available from many low- and middle-income countries might be mistakenly understood and research in this field is important in every country to determine risk factors and appropriate intervention strategies.

## Supplementary information

**Additional file 1.** Flow diagram of study selection using Scopus database.

**Additional file 2.** Keywords and phrases used in the search strategy.

## Data Availability

Not applicable.

## Abbreviations

BO: Burnout
CF: Compassion fatigue
WHO: World Health Organization
