# Supplementary material for: Research trends and scientific analysis of publications on burnout and compassion fatigue among healthcare providers
Source: J Occup Med Toxicol. 2020 Jul 13;15:23. doi: 10.1186/s12995-020-00274-z (PMC7356120; doi:10.1186/s12995-020-00274-z)
Supplement: Supplementary file 1 — Additional file 1. Flow diagram of study selection using Scopus database. [file 12995_2020_274_MOESM1_ESM.docx]

**Supplementary material 1**

**Research trends and scientific analysis of publications on burnout and compassion fatigue among healthcare providers**

Flow diagram of study selection using Scopus database

Number of documents on healthcare providers

**N= 3042488**

Number of documents on "burn out"/ "compassion fatigue"

**N= 13605**

**Exclude documents on medical students**

**N = 4416**

**Exclude documents published on 2020**

**N = 4819**

**Limit to journal research articles**

**N= 4665**

Number of documents on burn out/ compassion fatigue among healthcare providers

**N= 5212**
