# Supplementary material for: Research trends and scientific analysis of publications on burnout and compassion fatigue among healthcare providers
Source: J Occup Med Toxicol. 2020 Jul 13;15:23. doi: 10.1186/s12995-020-00274-z (PMC7356120; doi:10.1186/s12995-020-00274-z)
Supplement: Supplementary file 2 — Additional file 2. Keywords and phrases used in the search strategy. [file 12995_2020_274_MOESM2_ESM.docx]

**Supplementary material 2**

**Research trends and scientific analysis of publications on burnout and compassion fatigue among healthcare providers**

Database: SciVerse Scopus

Keywords and phrases used in the search strategy

| **Number of documents retrieved** | **Keywords used** | **Step** |
| --- | --- | --- |
| **13605** | ( title ( "burnout"  or  "burn - out"  or  "compassion fatigue"  or  "burning out"  or  "burn out"  or  "occupational stress"  or  "professional *stress"  or  "emotional *stress"  or  "emotional exhaustion"  or  "secondary trauma*"  or  "vicarious trauma*"  or  "psychological *stress" )  or  title ( empathy  or  "physical exhaust*"  or  depersonalization  or  "feeling* of cynicism"  or  detachment  or  "depletion of energy"  or  "well being" )  and  title-abs-key ( burnout  or  "compassion fatigue" ) ) | **#1** |
| **3042588** | (title ( health*  or  nurs*  or  physician  or  doctor  or  pharmacist  or  "healthcare"  or  "medical"  or  resident  or  clinician*  or  paramedic  or  hospital  or  "icu"  or  emergency  or  surgeon  or  orthopedic  or  residency  or  surgical  or  oncology  or  neurology  or  neurologist  or  "medicine"  or  oncologist  or  psychiatrist  or  midwife  or  clinic ) ) | **#2** |
| **5212** | Combine (#1 AND #2) | **#3** |
| **4819** | Exclude documents published on 2020 | **#4** |
| **4665** | Limit to journal research articles | **#5** |
| **4416** | Exclude documents with titles having the keyword "student*" | **#6** |
